# Sustainable court surfaces using emulsion-waste rubber cushions and acrylic-rice husk top coatings

**DOI:** 10.1038/s41598-025-10189-6

**Published:** 2025-07-15

**Authors:** Samir M. M. Morsi, Ahmed M. Khalil, Samir Kamel

**Affiliations:** 1 Polymers and Pigments Department, National Research Centre, Giza, 12622 Egypt; 2 Photochemistry Department, National Research Centre, Dokki, Giza, 12622 Egypt; 3 Cellulose and Paper Department, National Research Centre, Dokki, Giza, 12622 Egypt

**Keywords:** Court surfaces, Cushioned coat, Acrylic emulsion, Styrene butadiene rubber, Mechanical properties, Pollution remediation, Polymers

## Abstract

This study explores a sustainable court surface system using styrene acrylic emulsion (SAE) and styrene butadiene rubber (SBR) binders combined with waste rubber powder (WRP) to develop cushioning layers that reduce surface impact. A pure acrylic top coat, reinforced with rice husk (RH), was formulated to improve slip resistance, abrasion durability, and UV stability on the cushions. A comparative analysis evaluated how filler amount, WRP proportion, and binder type and content influence the physical, mechanical, thermal, and morphological properties of the cushions. Increasing SAE or SBR content in cushions by 8.21% enhanced their tensile strength by 30.28% and 32.77%, respectively, due to improved matrix cohesion. However, the microporous structure of WRP introduced voids that reduced tensile strength and elongation, particularly at high loadings or when binder content was low, reflecting a trade-off between mechanical performance and shock attenuation. CaCO_3_ filler occupies voids within WRP, enhancing dimensional stability but disrupting polymer chain continuity and flexibility. This reduces free volume and limits chain mobility, both essential for elastic deformation. 15% RH-loaded topcoat reduced adhesion pull-off and elongation by 5.15% and 89.1%, respectively, while increasing tensile strength and abrasion resistance by 46% and 30.5%. These enhancements are attributed to the fibrous nature and filler interaction of RH within the acrylic matrix. The developed multilayer system is suited for indoor and outdoor multipurpose sports courts; such as those used for basketball and tennis where durability, shock absorption, and environmental sustainability are essential.

## Introduction

Court surfaces vary widely depending on the type of sport and environmental conditions. Each sport, whether individual or team-based, has specific requirements that influence the choice of flooring material^1–7^. For instance, natural grass is typically used in football fields and comprises a soil base with an upper layer of grass. Artificial turf, usually made from polyethylene, features a backing layer with synthetic fibers sewn into it. Tartan tracks consist of rubber granules bound with chemicals. Maple wood floors, known for durability and aesthetics, are preferred for indoor basketball courts but require costly maintenance^8–11^. Vinyl flooring, while inexpensive and common in gyms, tends to wear quickly. Parquet floors, common in squash courts, are installed over cement with a soft underlayer to support player movement. Foam flooring is primarily used in nurseries and schools for safety due to its softness. Rubber flooring is often found in weight rooms and gyms, offering multi-layer durability and excellent shock absorption^12,13^. Among all, acrylic courts are particularly versatile and widely used for sports such as tennis, handball, basketball, and multi-purpose activities, including in schools, hospitals, and playgrounds^11^. These floors consist of two main layers: an inner cushioning layer composed of rubber mixed with acrylic for shock absorption, and an outer slip-resistant, antimicrobial, and easy-to-clean acrylic top layer. The flooring is applied over cement concrete sealed with an acrylic primer and re-surfacer to ensure uniformity and durability^14,15^. The cushion acrylic layer, which incorporates rubber granules, adds flexibility and resilience, making these courts long-lasting, cost-effective, and suitable for both indoor and outdoor use.

In terms of sustainability, rice husk (RH) is an abundant agricultural byproduct in Egypt, with over 6 million tons generated annually. Much of it is incinerated, causing severe environmental and health issues. RH has potential applications in various industries such as wood-plastic composites, silica production, fertilizers, and as reinforcement in coatings. In this study, RH is utilized as a filler in the acrylic top coat to improve abrasion and slip resistance^16–20^. Likewise, waste rubber from damaged car tires poses a major environmental problem. Often discarded or burned, these materials are rich in potential for reuse. Ground rubber is currently used in rubber tiles, brake pads, asphalt, and insulation materials^21–25^.

Although acrylic sports flooring is widely used, there has been limited research on incorporating waste rubber powder (WRP) and rice husk (RH) into these systems with water-based binders like styrene butadiene rubber (SBR) and styrene acrylic emulsion (SAE). Most existing formulations still depend on virgin materials such as EPDM, which contribute to higher costs and environmental impact. Strąk et al.^26^ examined sports surfaces made from recycled SBR and EPDM, highlighting key mechanical and safety properties including shock absorption and wear resistance, which are essential for sports court performance. Xie and Huang^27^ investigated the aging behavior of EPDM, focusing on surface roughness changes and their effect on the durability of gasketed joints. Although their study centers on sealing applications, the insights gained are applicable to the longevity and surface quality of EPDM in sports flooring. Monaghan and Marshall^28^ evaluated an advanced crosslinking acrylic polymer aimed at enhancing abrasion resistance and water resistance in court coatings. Furthermore, Nobili et al.^29^ developed a multi-layer system that integrates a water-based acrylic resin, a fiber-reinforced PVC mat, and a removable adhesive, engineered for high wear resistance in professional tennis courts and indoor multi-sport venues.

This study aims to fill this gap by developing a sustainable, cost-effective cushioned acrylic court system incorporating WRP and RH, while evaluating the influence of filler content, rubber powder percentage, and binder type and content on mechanical, physical, thermal, and morphological properties. In addition, the work investigates RH as a functional additive in the acrylic top coat to improve slip resistance and abrasion durability. The results offer a novel, eco-friendly court surfacing solution with enhanced performance and reduced environmental impact, contributing to sustainable materials development in sports surface applications.

## Experimental

### Materials

Styrene (St), Methyl methacrylate (MMA), butyl acrylate (BA), and acrylic acid (AA) were obtained from Sigma-Aldrich. Acrylamide (AAm), sodium bicarbonate (NaHCO_3_), ammonia, and potassium persulfate (KPS) were supplied from Sigma Chemicals. Ionic and non-ionic surfactants such as Texapon N70 and nonylphenolethoxylate (NP30), respectively was supplied by BASF Company. Styrene butadiene rubber (SBR) latex is carboxylated styrene butadiene copolymer latex supplied from Euclid Chemical. Waste rubber powder (WRP) was provided from worn-out vehicles tires in Cairo, Egypt. The consumed tires were sliced to tiny segments. Thereafter, they were ground forming a powder. Rice husk was provided from El-Sharqia-Egypt. Calcium carbonate was purchased from the Egyptian Carbonate Co. for Mining-Egypt.

### Synthesis of styrene-acrylate and pure acrylate emulsions

Two types of emulsions; namely styrene-acrylate emulsion (SAE) and pure acrylate emulsion (PAE) were synthesized via semicontinuous seed emulsion polymerization^30,31^. A monomer mixture containing 102.5 g of butyl acrylate (BA) and either 83.3 g of styrene (St) for SAE or 50.1 g of methyl methacrylate (MMA) for PAE was emulsified in 80 mL of distilled water with 7 g of Texapon N70 using a homogenizer. The emulsification process was carried out at room temperature for 30 min. Separately, 10 wt% of the pre-emulsified monomer mixture was transferred to a reaction flask containing 100 mL of distilled water, 4 g of NP30 (non-ionic surfactant), 0.5 g of sodium bicarbonate (NaHCO_3_), and 0.1 g of potassium persulfate (KPS) as an initiator. The reactor was equipped with a condenser, mechanical stirrer, and vacuum pump, and maintained at 80 °C using a heating mantle. The seed formation was allowed to proceed for 30 min.

Subsequently, 6.5 g of acrylic acid (AA) and 2.3 g of acrylamide (AAm) were added to the remaining pre-emulsion. An initiator solution was prepared by dissolving 0.4 g of KPS in 35 mL of distilled water. Both the remaining pre-emulsion and the initiator solution were gradually added to the reactor over 4 h while maintaining constant stirring at 80 rpm. After the addition, the reaction was continued under vacuum at 85 °C for an additional 30 min. Finally, the pH of the resulting emulsions (SAE and PAE) was adjusted to 8 using ammonia solution during the cooling process.

### Formulations of cushion layers

A stainless steel 20-mesh sieve (800 μm) was used to analyze the particle size distribution of the waste rubber powder (WRP). The fraction that passed through the sieve was used in subsequent formulations. Predetermined amounts of WRP, calcium carbonate (CaCO_3_), and either styrene-acrylate emulsion (SAE) or styrene-butadiene rubber (SBR) latex were mixed in a beaker using an Ultra-Turrax homogenizer for 5 min at ambient temperature. The detailed composition of the cushion layer formulations is presented in Table 1.

**Table 1:** Composition of cushion layer formulations using SAE and SBR binders (values in parts by weight).

Formulation code	WRP	CaCO_3_	SAE	SBR
CA1	40	20	50	–
CA2	50	20	50	–
CA3	60	20	50	–
CA4	50	10	50	–
CA5	50	30	50	–
CA6	50	30	60	–
CA7	50	30	70	–
CS1	40	20	–	50
CS2	50	20	–	50
CS3	60	20	–	50
CS4	50	10	–	50
CS5	50	30	–	50
CS6	50	30	–	60
CS7	50	30	–	70

*CA* Cushion formulation using SAE, *CS* Cushion formulation using SBR, *WRP* Waste rubber powder, *CaCO*_*3*_ Calcium carbonate, *SAE* Styrene-acrylate emulsion, *SBR* Styrene-butadiene rubber latex.

### Formulations of top coats

A 20-mesh (800 μm) stainless steel sieve was used to analyze the particle size distribution of rice husk (RH), and the fraction that passed through the sieve was used in the top coat formulations. Pure acrylate emulsion (PAE) was employed as the primary binder at 70% by weight of the formulation. Calcium carbonate (CaCO_3_) and red iron oxide pigment were each incorporated at 10% by weight. The remaining 10% comprised distilled water containing 0.6% of a dispersing agent, along with antifoam and a preservative. This base formulation was designated as TC0. To investigate the effect of RH content, RH was added to TC0 at 5%, 10%, and 15% by total weight of the formulation, yielding modified top coats labeled as TC5, TC10, and TC15, respectively.

### Characterization methods

The solid content (S.C.) of all formulations was determined as the average of three experiments, following *ASTM D4209-07*. Viscosity was measured according to *ASTM D2196-10* using a Brookfield Digital Viscometer DV-E (spindle #7, 5 RPM). Density was measured based on *ASTM D1475-13* using a 100 mL stainless steel pycnometer. The consistency of the formulations was determined using a Sheen viscometer in accordance with *ASTM D562*.

Abrasion resistance of the top coats was evaluated using a Taber Abraser Tester Model 5150 according to *ASTM D4060-19*. Coated samples were subjected to 1000 cycles using non-resilient wheels (CS-10, CS-17, and H-22), and the weight loss was recorded in milligrams. Pull-off adhesion of the coating systems on concrete substrates was assessed in accordance with *ASTM D4541-22*. A PosiTest Pull-Off Adhesion Tester applied force to a 20 mm dolly bonded to the surface. Flexibility and resistance to cracking or detachment from metal substrates were also assessed using a Sheen Cylindrical Mandrel Bend Tester (mandrel diameter: 2 mm), in accordance with *ASTM D522*. UV aging tests were conducted to assess the impact of UV light on the mechanical properties of the top coats, following *ASTM D4587-91*. Samples were exposed to 4 W, 312 nm UV lamps at room temperature for 300 h. The opacity and shade of the top coats were measured using a Novo-Shade Duo+, a portable 45°/0° reflectometer. Opacity was evaluated by applying coatings onto Leneta Form 10B sealed checkerboard charts. Specular gloss at a 60° angle was measured using a Minigloss 60° Glossmeter, model 101 N (Sheen Instruments, UK), in accordance with *ASTM D2457*. Gloss values represent the average of ten readings. Impact resistance was determined using a 2 kg tubular impact tester as per *ISO 6272-2*. The weight was dropped from a height of 90 cm onto the coated surface facing upward (intrusion). Scratch resistance of the top coats was evaluated using a PUSHEN ZHY automatic scratch tester. The test was performed with a 10 cm scribing needle at a traverse speed of 3 cm/s under increasing loads (100–2000 g). The critical load at which a visible scratch occurred was recorded. Pencil hardness was measured following *ASTM D3363* using a Wolff-Wilborn pencil tester. Pencils were held at a 45° angle, and hardness was defined as the hardest pencil that caused a surface scratch.

Thermal stability was evaluated by thermogravimetric analysis (TGA) using a Shimadzu TGA-50 (Shimadzu, Columbia, USA) at a heating rate of 10 °C/min from room temperature to 600 °C. The samples were die-cut into dumbbell shapes to evaluate their mechanical properties, including tensile strength and elongation at break, in accordance with *ASTM D412-16*. Measurements were performed using a Zwick universal testing machine, with three replicates conducted for each sample. The particle size and distribution of the emulsions were measured by dynamic light scattering (DLS) using a Malvern Zetasizer Nano (Malvern Instruments, UK) over a range of 0.4–10,000 nm. The reported particle size values represent the average of three independent measurements. Differential scanning calorimetry (DSC) was performed using a DSC Q2000 V24.11 Build 124 (TA Instruments) at a heating rate of 10 °C/min in the range of − 80 °C to 100 °C. Scanning Electron Microscopy (SEM) was performed using a Quantum Field Emission Gun 250. Surface topography was investigated using atomic force microscopy (AFM) with a WiTec alpha 300R Raman Imaging Microscope. Images were captured in non-contact mode using a silicon cantilever (280–300 kHz, 42 N/m). Depth profiles were acquired in contact mode using a silicon cantilever with a 0.2 N/m force constant.

## Results and discussion

### Properties of emulsions

Table 2 presents the key physical and chemical properties of the emulsions used as binders in the formulations. SAE and PAE exhibit higher solids and binder contents compared to SBR, which also contains a minor ash content of 1.53%, whereas SAE and PAE contain none. The substantial differences in viscosity among these emulsions are primarily due to variations in their molecular structures, particle sizes, and solids contents. SAE and PAE, both acrylic-based emulsions, show significantly higher viscosities (8300 cP and 7855 cP, respectively) as a result of their higher molecular weights, denser cross-linking, and narrower particle size distributions. In contrast, the lower viscosity of SBR (220 cP) is attributed to its lower solid content and broader particle size distribution, which reduce internal friction and flow resistance.

**Table 2:** Properties of emulsions used in the formulations.

Property	SAE	PAE	SBR
Solid content (S.C.), %	52.12	51.32	48.41
Binder content, %	52.12	51.32	46.88
Ash content, %	–	–	1.53
Viscosity (Sp4 / 20 RPM), cP	8300	7855	220
Consistency, k	109.7	107.2	54.8
Density, g/cm^3^	1.0477	1.0394	1.0145
pH	8	8	10
Particle size distribution (PSD), nm	122	86	120
Polydispersity index, PDI	0.108	0.112	0.121

**Fig. 1:**
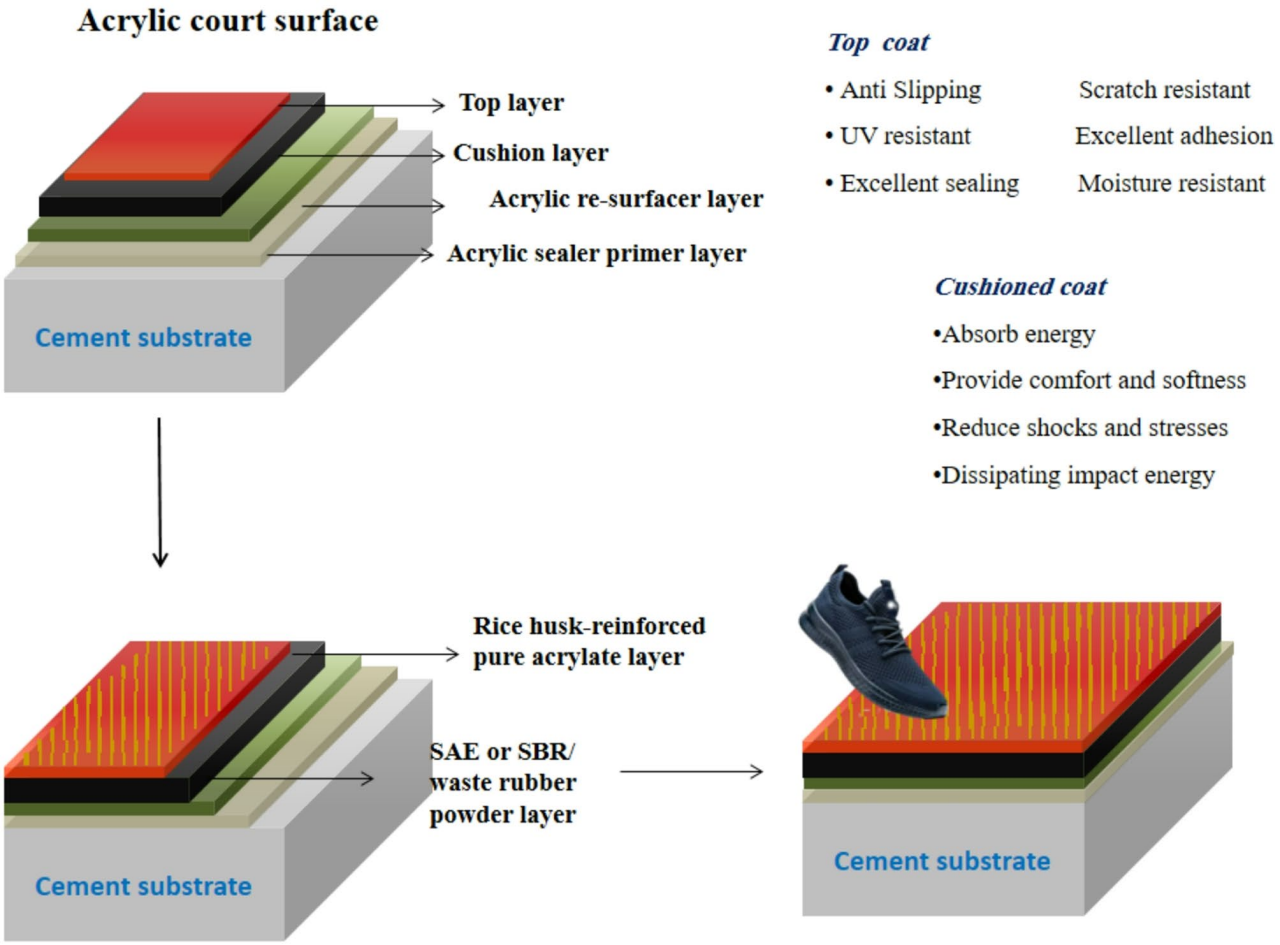
A schematic diagram for the different polymeric layers coated with the developed top layer of this work (in orange).

Viscosity plays a vital role in determining the rheological behavior and application performance of the formulations. Higher viscosity contributes to improved suspension stability of fillers such as RH and WRP, minimizing settling and ensuring uniform coating thickness during application. In the cushion layers, it also enhances the material’s ability to conform to surface irregularities, which is important for achieving optimal mechanical performance and shock absorption. Overall, SAE and PAE emulsions demonstrate superior viscosity, consistency, and density compared to SBR, making them more suitable for achieving uniform and stable coating formulations. Figure 1 displays an illustration for the various polymeric layers designed and applied including the cushion layers and coated with the developed orange top layer of this work. This figure has been drawn by using PowerPoint 2016 software.

### Physicochemical properties of cushions

**Fig. 2:**
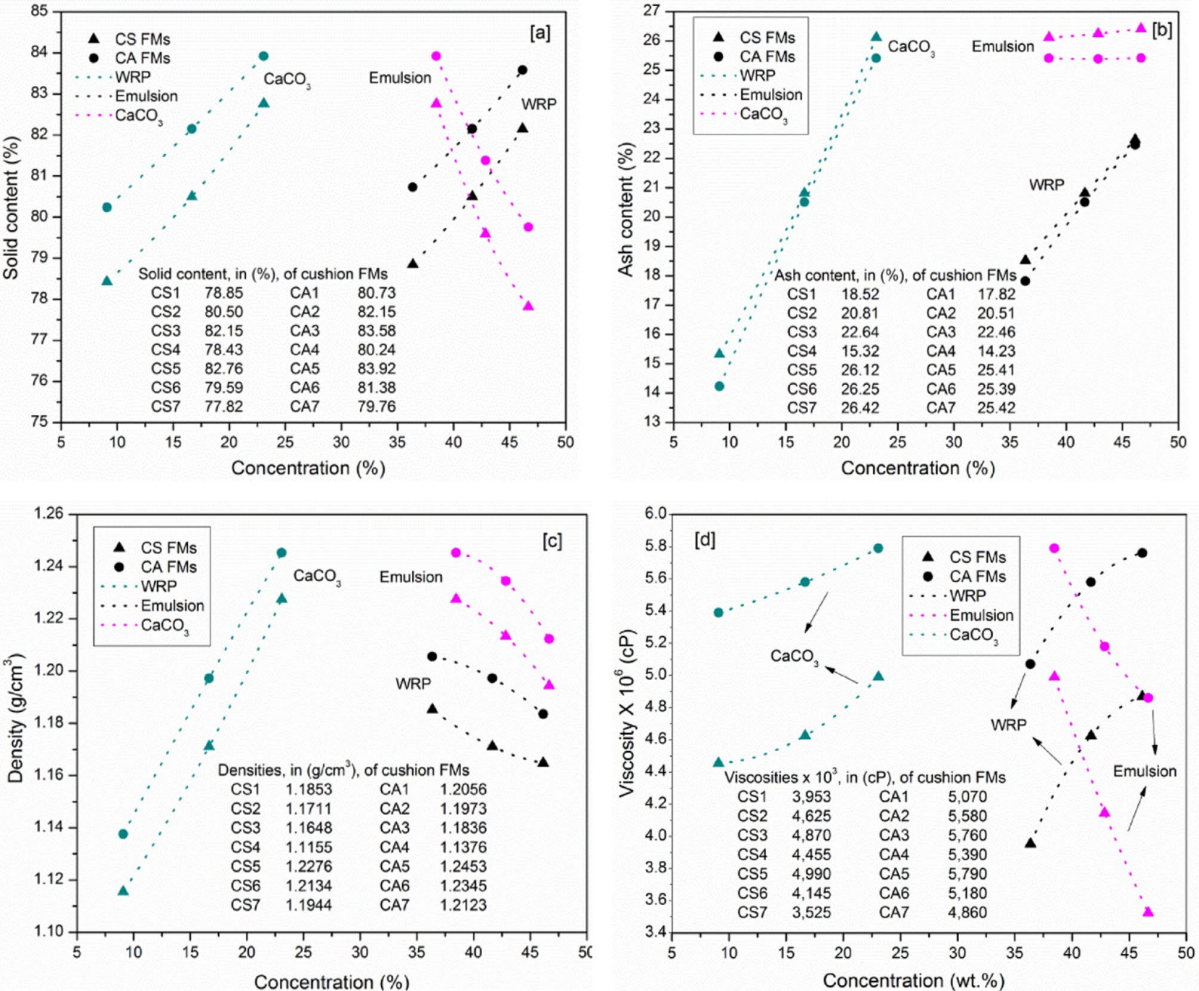
Effect of concentration of filler, emulsion, and WRP on the solid content (**a**), ash content (**b**), density (**c**), and viscosity (**d**) of cushion formulations.

Figure 2a illustrates the influence of varying concentrations of key formulation components of CaCO_3_, emulsion, and waste rubber powder (WRP) on the solid content of cushion formulations (CS and CA). The solid content increases with higher concentrations of CaCO_3_ filler and rubber powder, while it decreases as the emulsion content (SBR or SAE) rises. This trend is expected, as emulsions contain a solvent, meaning greater water presence increases volatile matter while reducing the solid fraction. Consequently, formulations with higher emulsion content exhibit potential dilution effects, limiting structural integration at higher concentrations. In contrast, CaCO_3_ and WRP promote a progressive rise in solid content, suggesting improved material stability and efficient filler incorporation. The highest solid content is observed in formulations CA3, CA5, CS3, and CS5, which contain the largest proportions of rubber powder (46%) and CaCO_3_ filler (23%). Additionally, SAE-based formulations consistently demonstrate higher solid content compared to their SBR counterparts, attributed to SAE’s naturally greater solids percentage (Table 2).

Figure 2b shows the ash residue of the samples after drying at 105 °C for 1 h to remove moisture, followed by combustion at 800 °C in a muffle furnace. The increase in ash content with higher filler levels is evident, primarily due to the decomposition of calcium carbonate, which releases CO_2_ and leaves behind calcium oxide. A similar trend is observed with increasing WRP content in the formulations, as the waste rubber powder was found to contain approximately 18% ash. This value was determined experimentally using the same combustion procedure, confirming that WRP contributes significantly to the residual inorganic content. Conversely, the emulsions (SAE and SBR) have minimal impact on ash content, remaining nearly constant across samples. However, SBR-based formulations exhibit slightly higher ash levels than those based on SAE, due to the inherent ash content in the SBR latex (1.53%, as shown in Table 2). As a result, CS7, which contains the highest proportions of both filler and WRP, displays the maximum ash content at 26.42%. Figure 2c illustrates the effect of formulation composition on the coating density. In general, SAE-based cushion coating formulations exhibit higher density than their SBR counterparts, attributed to the higher intrinsic density of the SAE emulsion (1.0477 g/cm^3^ vs. 1.0145 g/cm^3^ for SBR, as shown in Table 2). Increasing the filler content leads to a marked rise in density, while increasing the rubber powder or emulsion content results in a decrease. For instance, in CS and CA formulations, raising the filler content from 9 to 23% increased density by 10.05% and 9.47%, respectively. This effect stems from the high density of calcium carbonate (2.71 g/cm^3^) relative to other formulation components. As seen in Fig. 2d, both CaCO_3_ and WRP act as rheological modifiers. The increase in viscosity can be attributed to the physical interactions between CaCO_3_, WRP, and the polymer chains of SAE and SBR, which enhance the formulation’s viscosity. Specifically, viscosity increased by 12.01% and 7.42% with CaCO_3_ loading from 9 to 23%, and by 23.20% and 13.61% when WRP content increased from 36.36 to 46.15% in CS and CA formulations, respectively. Conversely, viscosity decreased with increasing emulsion content, which introduced more water and reduced the formulation’s resistance to flow. SAE-based mixtures consistently showed higher viscosity than their SBR counterparts, aligning with the higher viscosity of the SAE emulsion itself (Table 2).

### Mechanical properties of cushion sheets

**Fig. 3:**
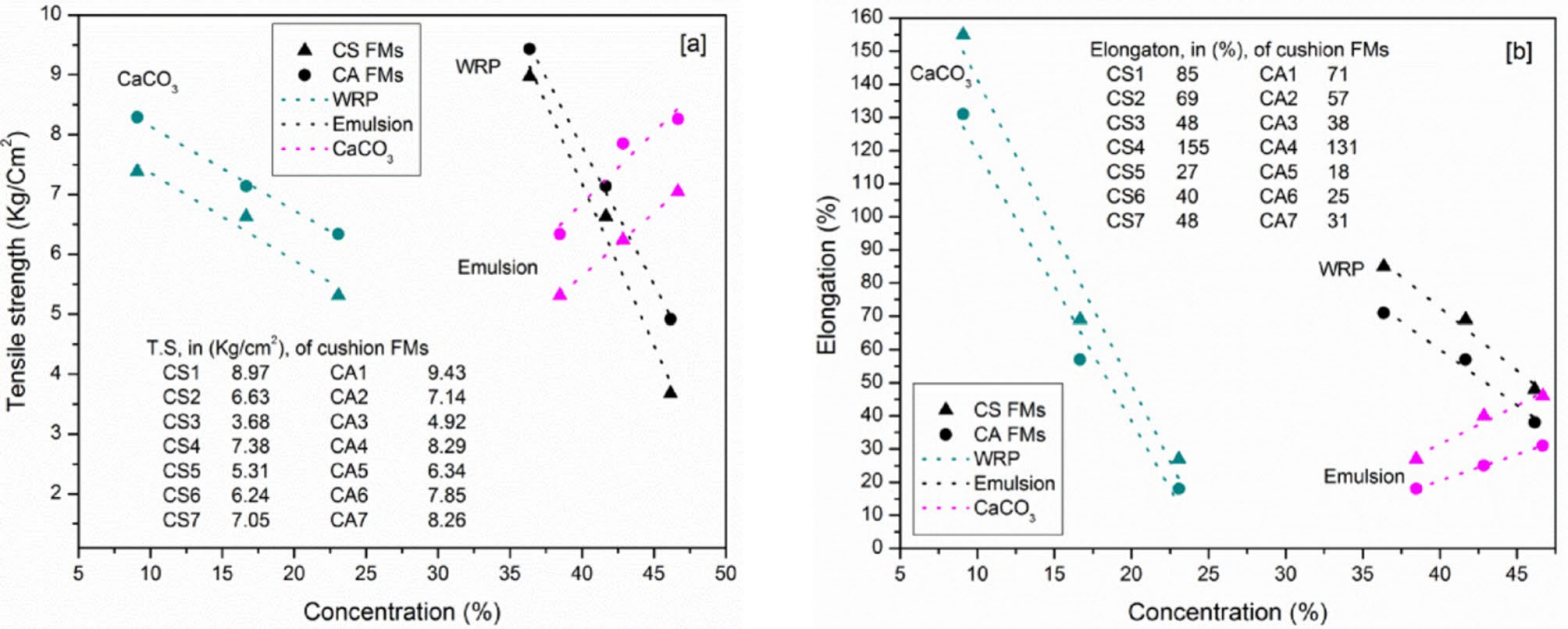
Tensile strength (**a**) and elongation (**b**) of the cushion layers. Numerical values shown within the graph (**a**) represents tensile strength in kg/cm^2^ (not density).

The tensile strength and elongation at break of the cushion sheets are presented in Fig. 3. As shown in Fig. 3a, tensile strength increases with higher binder content but decreases with increasing amounts of WRP and CaCO_3_ filler. Increasing the SBR and SAE from 38.46 to 46.67% in CS and CA sheets led to increases in tensile strength of 32.77% and 30.28%, respectively. Conversely, the addition of WRP and CaCO_3_ reduced elongation values (Fig. 3b), whereas increasing the binder content improved elongation by 77.78% and 72.22% in CS and CA sheets, respectively. In all formulations, the polymer chains form the matrix within which the filler and rubber powder are dispersed. The flexibility of the polymer chains and their entanglement contribute to greater elongation when binder content is higher. However, reduced binder content limits these interactions, decreasing the elongation capacity. Physical interactions between the entangled polymer chains and the filler materials (WRP and CaCO_3_) increase the force required to pull the sheets, contributing to the tensile strength. Notably, the use of rubber in powdered form, rather than as a continuous molten phase, enhances elasticity but reduces both tensile strength and elongation due to weak interfacial bonding. Regarding binder type, SAE-based sheets demonstrated higher tensile strength than their SBR counterparts (Fig. 3a), whereas SBR-based sheets exhibited greater elongation at break (Fig. 3b). This behavior is attributed to the chemical structure of the binders: SAE contains functional groups that facilitate network formation with both itself and fillers, enhancing tensile strength. In contrast, SBR chains lack such functional groups but offer greater flexibility, resulting in higher elongation. The presence of CaCO_3_ in the cushion formulation enhanced dimensional stability and density but adversely affected the elasticity of the material. As a rigid, non-deformable filler, CaCO_3_ occupied the voids within the rubber powder and restricted the mobility of the polymer chains. It did not contribute to elastic deformation or energy dissipation; instead, it reduced the flexibility of the polymer matrix by limiting the free volume for chain movement and disrupting the continuity of the elastomeric network. This led to stiffer and less elastic cushions, particularly when CaCO_3_ was used in high proportions or when the binder content was insufficient to preserve chain entanglement and matrix cohesion.

### Morphological characterization of the cushions

**Fig. 4:**
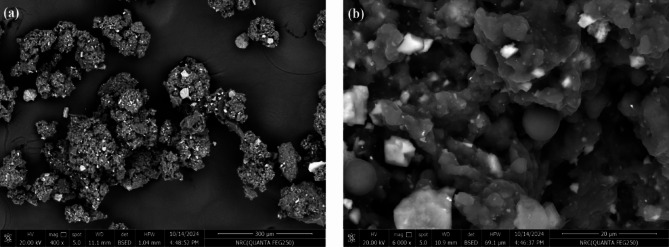
SEM images WRP at magnification of ×400 (**a**) and ×6000 (**b**).

Figure 4a shows the SEM image of WRP at 400× magnification, illustrating the particle size distribution of material sieved to below 800 microns. The image predominantly shows particles smaller than 500 microns with irregular shapes and a void-rich structure. Figure 4b provides a closer look at 6000× magnification, revealing a porous, flake-like texture interspersed with rounded grains. The images indicate a highly uneven surface topography with dispersed voids and structural irregularities, suggesting a large specific surface area. This increased roughness and porosity may enhance interfacial adhesion when WRP is incorporated into coatings or composite matrices. Additionally, the observed heterogeneity in particle shape and size, along with the presence of interconnected structures, implies either particle agglomeration or inherent porosity within the material.

**Fig. 5:**
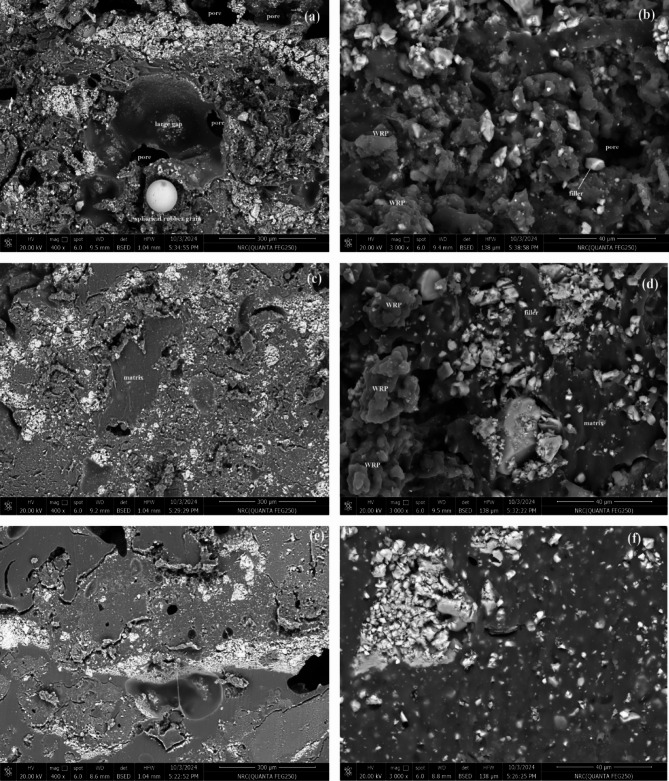
SEM images CA3 (**a**,**b**), CA5 (**c**,**d**), and CA7 (**e**,**f**) of cushion layers at magnification of ×400 and ×3000, respectively.

**Fig. 6:**
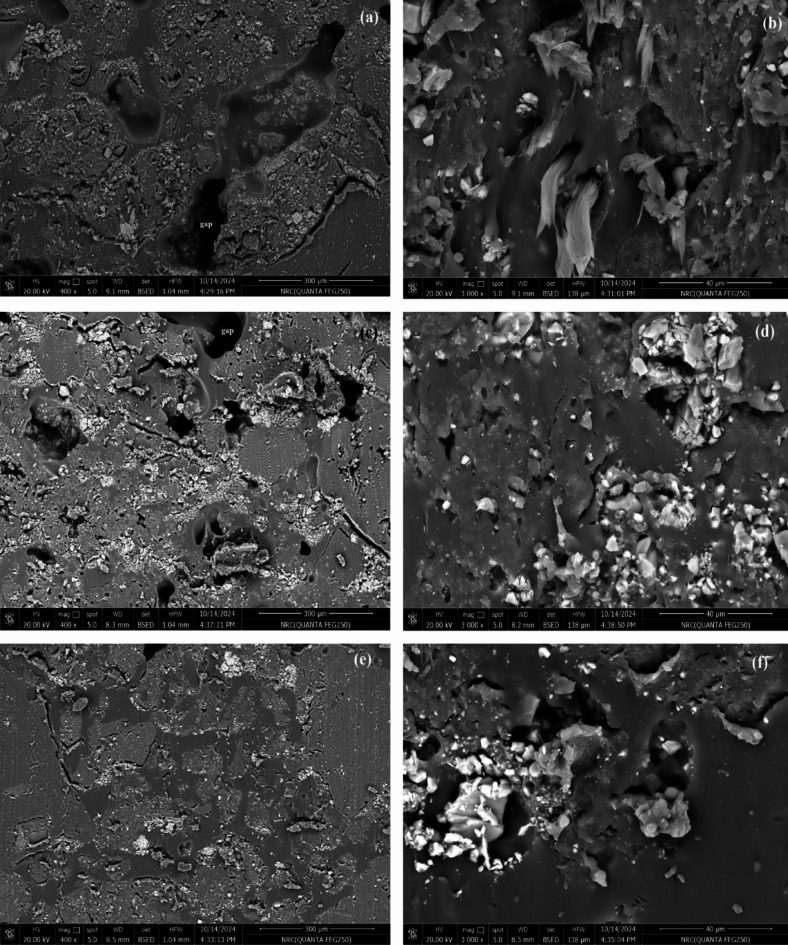
SEM images CS3 (**a**,**b**), CS5 (**c**,**d**), and CS7 (**e**,**f**) of cushion layers at magnification of ×400 and ×3000, respectively.

Figures 5 and 6 present SEM micrographs of selected cushion coatings at magnifications of 400× and 3000×. Figure 5a,b present SEM images of the CA3 cushion layer at magnifications of 400× and 3000×, respectively. The micrographs reveal a heterogeneous microstructure characterized by numerous pores and voids, approximately 50–150 μm in size, distributed throughout the matrix. The irregular morphology and dispersed voids suggest incomplete dispersion, agglomeration, and poor interfacial bonding. These features contribute to compressibility and cushioning. The observed porosity aligns with the role of WRP and CaCO_3_ as rheological modifiers affecting density and mechanical performance. Figure 6a,b illustrate the corresponding sample, CS3, which displays large voids and high porosity, accompanied by structural cracks. At lower magnification (Fig. 6a), large voids and embedded WRP are visible, indicating possible air entrapment and non-uniform dispersion. The higher magnification (Fig. 6b) reveals rough, flaky features and microcracks, suggesting weak interfacial bonding between the matrix and fillers. These morphological characteristics may affect mechanical performance and compressibility of the cushion. Under tensile stress, these defects accelerate crack propagation, ultimately diminishing mechanical properties. However, these voids provide space for rubber contraction upon vibration, contributing to effective shock attenuation.

The voids within the cushion layers are reduced with increased CaCO₃ content, as observed in CA5 and CS5 (Figs. 5c,d and 6c,d). The microstructure exhibits a dense, heterogeneous network with fewer interfacial defects. This is due to the effective binding of fillers to rubber powder by SAE and SBR matrices. While the matrix partially encapsulates the fillers, some remain exposed. This structure results in a rough, porous morphology that contributes to reduced elasticity as filler content increases. With the increase of the binding material, CA7 and CS7 (Figs. 5e,f and 6e,f), the polymer matrix becomes more dominant, leading to a more compact and uniform structure. In these micrographs, the surface morphology appears smoother, with SAE and SBR effectively integrating all components, including fillers and WRP. This results in improved tensile strength and greater elongation capacity of the cushions.

### Thermal analysis of the cushions

**Fig. 7:**
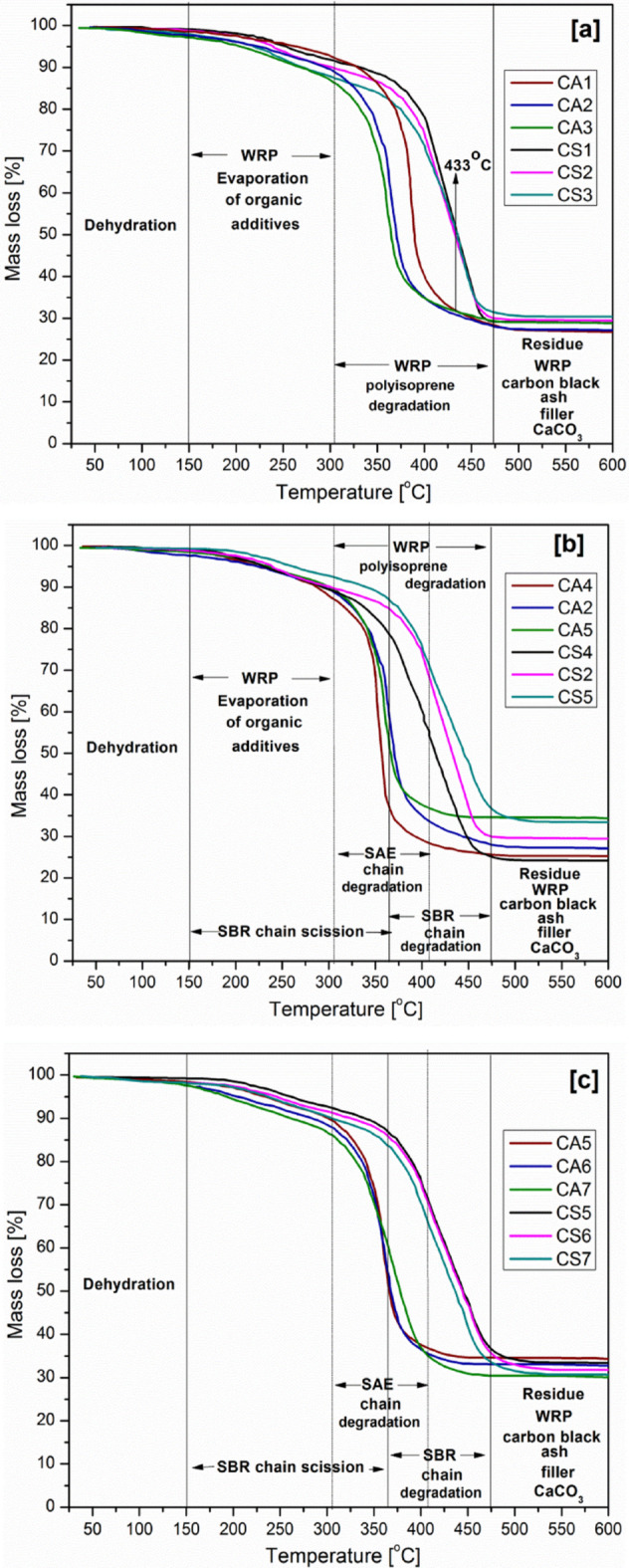
TGA thermal curves of cushion coats: (**a**) CA and CS [1, 2 and 3], (**b**) CA and CS [2, 4 and 5] and (**c**) CA and CS [5, 6 and 7].

The TGA profiles of the cushion layer samples are displayed in Fig. 7. To examine the impact of varying the type of binder from SAE to SBR and the quantity of WRP, filler, and binder on the thermal characteristics, the curves were distributed into three images. In general, it is evident that SBR samples have more stable thermal stability than their SAE counterparts. The impact of WRP content on the cushion layer’s thermal stability is depicted in Fig. 7a. The WRP is assumed to decompose in several steps, as reported in literature^32^. The first step, from room temperature up to 150 °C, is due to moisture evaporation. The next stage, up to ~ 300 °C, corresponds to the loss of low molecular weight rubber additives, such as oils and plasticizers. Pyrolysis of elastomeric components; typically polyisoprene or butadiene rubber in tire-derived powders is reported to occur above 300 °C, leaving behind a residue primarily composed of carbon black and mineral fillers such as silica. Thus, it is clear from the image that the thermal stability of the samples decreases with increasing amount of rubber powder up to 433 °C in both groups (CA and CS), due to the decomposition of polyisoprene groups in them. Then the thermal stability reverses after this temperature because the remaining rubber of carbon black and ash is more in quantity. Thus the residue at 600 °C is 26.7% for CA1 (the least in WRP), which increases to 27.1% and 29.7% for CA2 and CA3 (the most in WRP), respectively. Similarly, the residue increases from 28.9% (CS1) to 29.4% and 30.3% for CS2 and CS3, respectively. This remainder represents carbon black, ash, and CaCO_3_. Also, the residue in SBR samples is slightly larger than their SAE counterparts due to the lower solid content of SBR compared to SAE and the inclusion of ash in SBR. As shown in Fig. 7b, with the increase in the percentage of filler, the thermal stability of the samples increases in all temperature ranges for the samples CA and CS (4, 2 and 5). As for the effect of the binder content, it is noted that with the increase in the binder amount, the thermal stability decreases. SAE chains begin to thermally degrade at a temperature of 311 °C, where the ester groups decompose as illustrated in Fig. 7c upon expressing the samples CA and CS (5, 6 and 7). SBR thermally decomposes in two stages. The instability of double bonds in SBR causes first reactions including chain scission, chain crossing, and oxidation to occur at about 200 °C and the second at around 370 °C. The initiation of free radicals generates allylic macroradicals, which are oxidized quickly into hydroperoxide species. The latter decomposes rapidly into carbonyl groups, ketones, aldehydes, esters, alcohols, and other oxides. During this period, crosslinking reactions occur between them^33^.

### Physicochemical and optical characteristics of the topcoat formulations

Table 3 summarizes the comprehensive physicochemical, optical, and film performance properties of the top coat formulations (TC0 to TC15). As the RH content increased, a gradual rise in solid content (S.C.%) was observed, from 55.74% in TC0 to 59.75% in TC15, accompanied by a decrease in binder content, reflecting the partial substitution of polymeric binder with RH filler. The viscosity increased significantly with RH loading, rising from 1552 cP in TC0 to 2844 cP in TC15. This suggests enhanced consistency and internal resistance to flow, likely attributed to the structural reinforcement and network formation introduced by the fibrous nature of the RH filler. In contrast, the density exhibited a slight decline, which is consistent with the lower intrinsic density of RH compared to the polymeric binder. In terms of appearance, both shade and gloss values decreased with increasing RH content, The reduction in gloss (from 60.5 to 32.1 about 47%.) and darker shade indicate that RH influences surface texture and light reflection. However, opacity, the measure of coat impenetrability to visible light, improved from 95.2 to 98.8%, indicating enhanced light-blocking capability which may benefit aesthetic coverage and UV shielding properties. Performance characteristics including UV resistance and touch-dry time remained within acceptable limits across all formulations. All samples successfully passed the UV resistance test (300 h), attributed to the use of a pure acrylate resin matrix known for its excellent photostability. The touch-dry time slightly decreased from 30 to 25 min with RH addition. These results highlight the robustness and environmental durability of the coatings, which are critical for ensuring the safety and longevity of playground and court surfaces.

**Table 3:** Physicochemical, optical, application/film properties of top coatings.

Property	TC0	TC5	TC10	TC15
S.C. %	55.74	57.33	58.50	59.75
Binder %	34.21	32.88	31.77	30.41
Viscosity, sp.5, 100 rpm (cp.)	1552	1948	2592	2844
Density	1.2621	1.2554	1.2516	1.2403
Shade	65.2	60.8	59.9	54.5
Opacity	95.2	96.3	98.5	98.8
Gloss	60.5	41.4	36.5	32.1
UV resistance, 300 h	Pass	Pass	Pass	Pass
Touch dry (min)	30	30	30	25

### Mechanical properties of the top coat formulations

Table 4 lists the mechanical and surface properties of the top coat formulations. Mechanical tests are essential for assessing the performance and surface characteristics of coatings^34–36^.

**Table 4:** Mechanical properties of top coatings.

Property		TC0	TC5	TC10	TC15
Impact resistance (drop weight 2 kg/90 cm)		Pass	Pass	Pass	Pass
Flexibility test (bend on 2 mm mandrel)		Pass	Pass	Pass	Pass
Scratch resistance (load in grams)		500	800	1000	1500
Pencil hardness		4B	H	2 H	3 H
Adhesion pull off strength (MPa)		2.33	2.31	2.28	2.21
Abrasion resistance (weight loss, mg)	CS-10	26.25	21.56	20.33	18.25
	CS-17	42.52	35.21	32.15	29.15
	H-22	85.23	77.20	73.12	68.12
Tensile strength (kg/cm^2^)		13.26	15.29	18.35	19.37
Elongation (%)		550	325	183	60

#### Impact and flexibility tests

Impact resistance evaluates a coating’s ability to absorb shock without cracking, while the flexibility test determines whether the coating can bend or stretch without breaking. Mechanical tests including impact resistance and bending flexibility were within acceptable limits across all formulations. Impact resistance was confirmed by each formulation’s ability to withstand the impact of a 2 kg weight dropped from a height of 90 cm, without exhibiting cracks or surface deformations. Additionally, all coatings demonstrated high flexibility, showing no signs of cracking or damage when bent around a 2 mm diameter mandrel^37^.

#### Scratch and hardness tests

Hardness and scratch tests measure a coating’s resistance to scratches and indentation. Notably, scratch resistance improved significantly rising from 500 g in TC0 to 1500 g in TC15. They highlight the reinforcing effect of RH in enhancing surface durability. This trend was further supported by an increase in pencil hardness, which shifted from 4B in the control sample to 3 H in TC15, indicating a substantial improvement in surface hardness^38^.

#### Adhesion pull-off strength

The adhesion test evaluates how well the coating adheres to a surface. While a slight reduction in adhesion pull-off strength was observed declining from 2.33 MPa in TC0 to 2.21 MPa in TC15. All values remained within acceptable ranges for protective surface coatings^39,40^.

#### Abrasion resistance

**Fig. 8:**
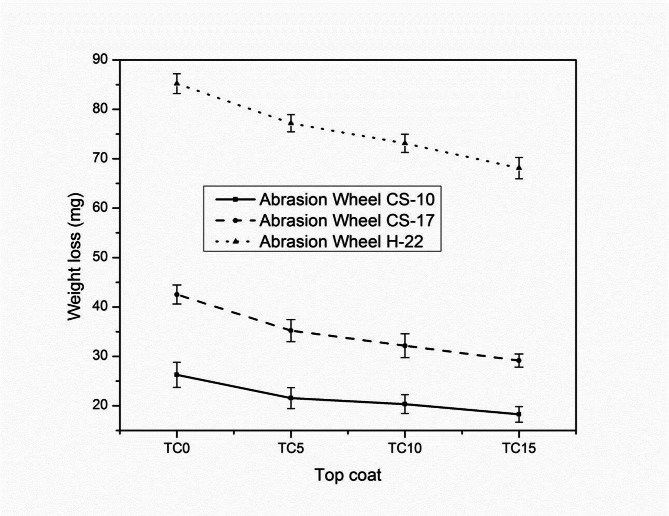
Abrasion resistance of top coats using CS-10, CS-17, and H-22 abrasive wheel.

The abrasion test evaluates a coating’s ability to withstand wear and friction over time. Figure 8 presents the results of the abrasion tests conducted on the prepared top coatings. In general, higher abrasive wheel numbers correspond to greater weight loss, indicating increased surface wear. It was observed that the incorporation of RH into the coating formulations significantly reduced the weight loss across all types of abrasive wheels. This suggests that RH enhances the surface hardness and roughness of the top layer, contributing to improved abrasion resistance. The inclusion of RH led to reductions in weight loss of up to 30.5%, 31.5%, and 20.1% for coatings tested with the CS-10, CS-17, and H-22 wheels, respectively, when comparing TC0 (control) to TC15 (highest RH content). These enhancements in abrasion resistance imply that RH-modified surfaces offer improved durability and friction, which are critical for athletic court applications. Such surfaces provide players with better traction and stability, thereby reducing the risk of slipping and potential injury^41^.

#### Tensile strength and elongation at break

**Fig. 9:**
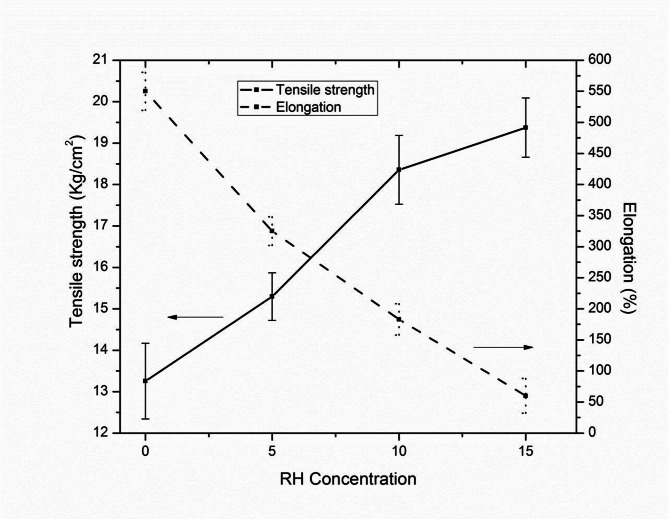
Effect of RH concentration on tensile strength and elongation of the top coats.

Figure 9 illustrates the influence of RH concentration on the tensile strength and elongation of the topcoats. A progressive increase in tensile strength was observed with rising RH content, from 13.3 kg/cm^2^ in TC0 to 19.4 kg/cm^2^ in TC15. This enhancement is attributed to the reinforcing action of RH constituents, particularly cellulose, hemicellulose and lignin. They promote to stronger interfacial bonding and more efficient stress transfer within the polymer matrix. The fibrous and rigid nature of RH contributes to this enhanced stress-bearing capacity, improving tensile strength by ~ 46%. Conversely, elongation at break decreased markedly from 550% in TC0 to 60% in TC15. The decline in elongation reflects reduced flexibility, a typical behavior in composite systems where the rigid and fibrous nature of RH restricts the mobility of polymer chains. The RH acts as a stiffening agent, which, while improving strength, limits the material’s ability to stretch without breaking. The error bars in the figure indicate low variability and confirm the statistical reliability of these trends. These results reflect a strength-flexibility trade-off often encountered in fiber-reinforced coatings. Therefore, optimizing RH content is essential to balance mechanical robustness and ductility, particularly for performance-critical applications such as sports flooring^42,43^.

The mechanical tests results collectively suggest that RH incorporation enhances mechanical strength, durability, and functional surface properties, without compromising essential coating attributes, making it a viable additive for high-performance, abrasion-resistant court surfaces.

### Thermal analysis of the top coats

**Fig. 10:**
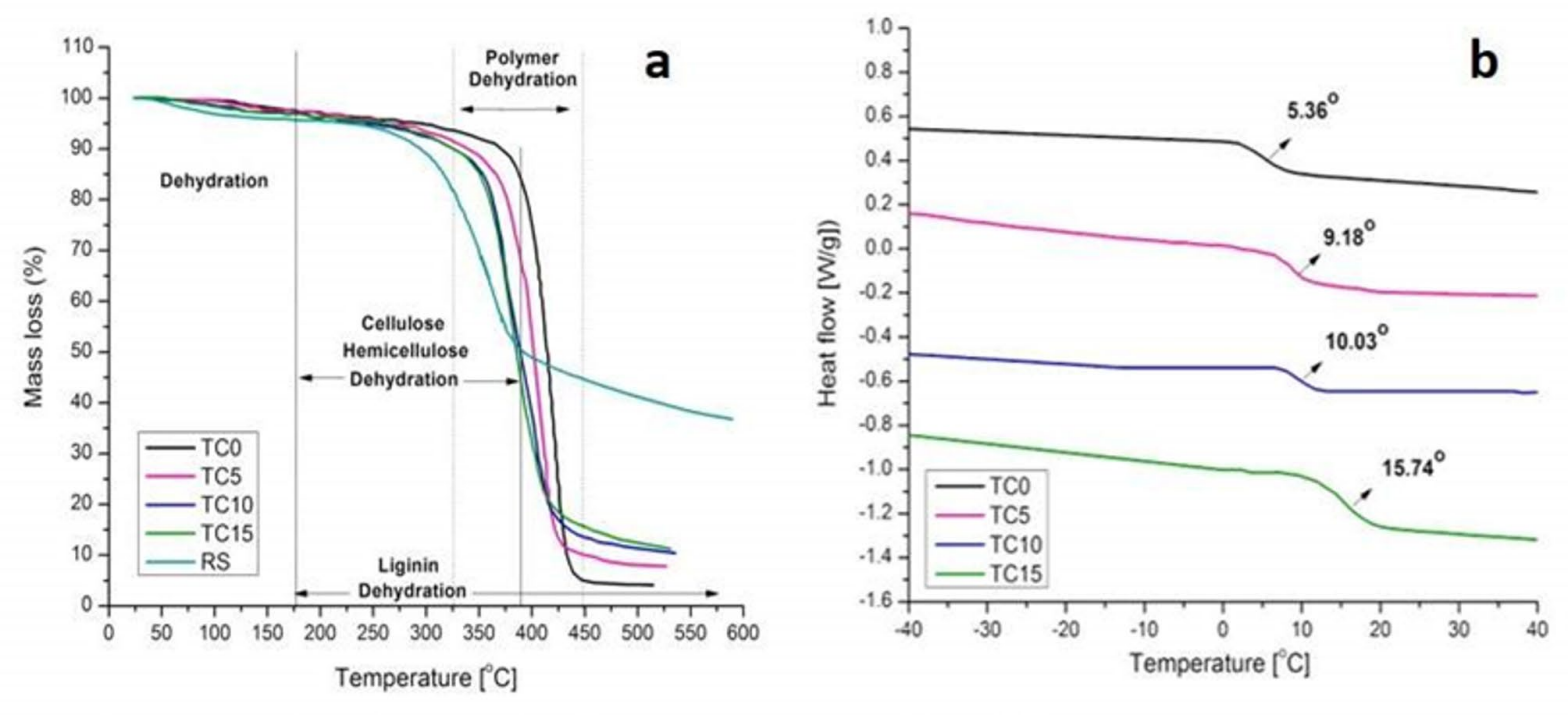
Thermal curves: (**a**) TGA and (**b**) DSC of rice straw and top coats.

Figure 10a shows the thermal decomposition profiles of RH and the prepared topcoat samples. The TGA curve of RH reveals three distinct regions: (i) moisture evaporation occurring between 30 °C and 110 °C, (ii) decomposition of hemicellulose and cellulose between 200 and 370 °C, and (iii) a broad, gradual degradation of lignin. The decomposition of hemicellulose and cellulose is attributed to hemicellulose’s low degree of polymerization and the disordered crystalline structure of cellulose. Lignin exhibits higher thermal stability, attributed to its aromatic structure and thermally stable functional groups. The ash residue yield from RH is approximately 37% of the initial mass.

For the unmodified top coat (TC0), a major weight loss step is observed around 410 °C, corresponding to the thermal degradation of acrylic polymer chains. Incorporation of RH into the top coats (TC5, TC10, and TC15) leads to reduced thermal stability, due to the decomposition of cellulose, hemicellulose, and lignin. However, RH addition increases the residual mass (ash content), indicating a higher inorganic or charred fraction in the RH-modified coatings.

Figure 10b presents the DSC thermograms of TC0, TC5, TC10, and TC15, highlighting their thermal transitions. A gradual increase in the glass transition temperature (Tg) is observed, from 5.36 °C (TC0) to 15.74 °C (TC15). This upward trend in Tg reflects restricted segmental motion of the acrylic chains, suggesting enhanced polymer–filler interactions and improved thermal stability. Additionally, prominent endothermic peaks are seen in all samples, representing thermal events associated with the acrylic matrix. The Tg shifts confirm increased rigidity in the RH-containing coatings, resulting in superior thermal resistance.

### Morphological characterization of the top coats

**Fig. 11:**
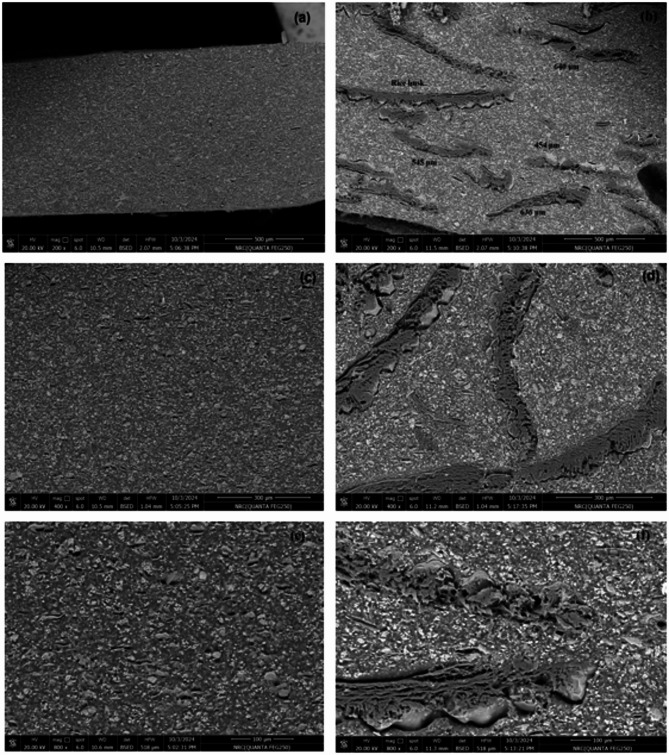
Cross-sectional SEM images if TC0 (**a**,**c**,**e**) and TC15 (**b**,**d**,**f**) at magnification of ×200 and ×400.

Figure 11 presents cross-sectional SEM images of TC0 and TC15 at magnifications of 200×, 400×, and 800×, illustrating their microstructural differences. Figure 11a,c,e show the micrographs of the unmodified top coat (TC0). These images reveal a polymer matrix with uniformly dispersed fillers and pigments, exhibiting a smooth, compact, and homogeneous structure with no visible agglomerations. In contrast, Fig. 11b,d,f display the microstructure of TC15, which incorporates RH. The RH fibers are randomly distributed within the matrix and predominantly exhibit a longitudinal orientation. Their lengths range from approximately 450–650 μm, with some exceeding 800 μm (Fig. 11b). The TC15 matrix appears more heterogeneous, with noticeable porosity and greater variation in structural features. At higher magnification (Fig. 11f), the RH fibers appear well-integrated into the matrix, functioning as embedded structural elements. This integration contributes to a rougher surface morphology compared to the smooth surface of TC0 and indicates a likely influence on the mechanical and textural characteristics of the coating. The structural differences between TC0 and TC15 clearly demonstrate the impact of RH incorporation on the microstructure. The increased fiber content and resulting heterogeneity in TC15 suggest potential improvements in tensile strength and enhance the coating’s ability to absorb shocks, offering functional advantages for impact-prone surfaces.

### Surface topography analysis of topcoats

**Fig. 12:**
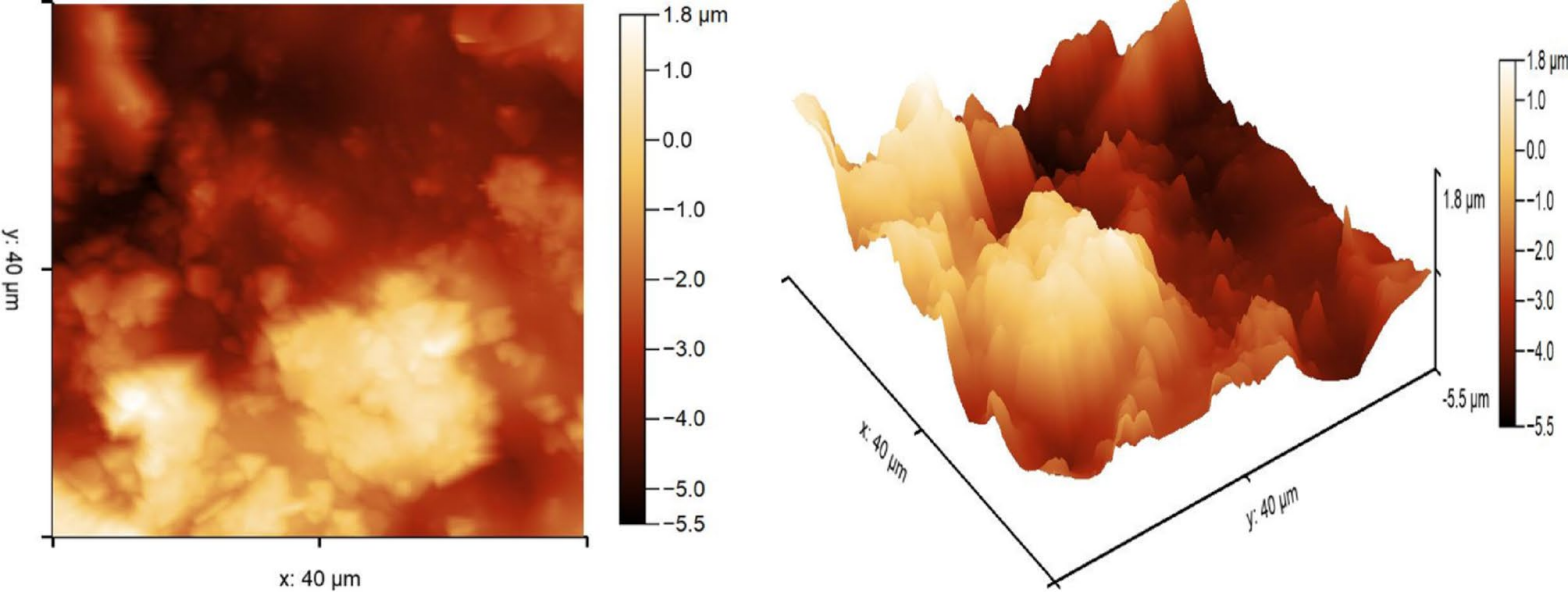
AFM 2D and 3D TC0 film.

2D and 3D Atomic Force Microscopy (AFM) images of the TC0 film are presented in Fig. 12. The images reveal a notably rough surface morphology, with pronounced surface irregularities characterized by elevated peaks and deep valleys—clearly visible in the 3D profile. The root mean square roughness (Rq) and arithmetic average roughness (Ra) were measured to be 1.53 μm and 1.27 μm, respectively. The surface topology further exhibits a maximum valley depth (Rv) of 2.97 μm and a maximum peak height (Rp) of 4.33 μm relative to the mean line, resulting in a peak-to-valley height (Rz) of 7.30 μm. The addition of rice husk (RH) to the top coat further increased the surface roughness. However, due to the extreme topographical features observed in RH-containing films, AFM measurements were discontinued to prevent potential damage to the cantilever probe. The pronounced roughness of the coating surface is expected to enhance slip resistance, which is a desirable property for safety in court and playground flooring applications.

## Conclusion

This study aimed to develop cushioned and top-layer coatings for durable and sustainable court surfaces. The cushion layers, composed of styrene acrylic emulsion (SAE) or SBR supported by waste rubber powder (WRP), exhibited a balance between mechanical strength and comfort. SAE-based cushions provided higher tensile strength, while SBR-based ones offered superior elasticity. Increasing WRP content improved cushioning (comfort) through enhanced softness and shock absorption, despite a moderate reduction in mechanical strength and thermal stability. Thermal analysis revealed that CaCO_3_ filler improved thermal stability, while binders had the opposite effect.

For the top coatings, RH significantly enhanced surface hardness, abrasion resistance, tensile strength, and surface roughness, though higher concentrations led to slight reductions in adhesion and flexibility. The integration of RH into acrylic formulations increased thermal stability by restricting polymer chain mobility.

These findings highlight the potential of the formulated coatings for performance-oriented court applications, combining durability, environmental resistance, mechanical integrity, and surface protection. The use of agricultural and rubber waste adds sustainability value, making these materials suitable for modern athletic flooring systems.

## Data Availability

Data availability: All data generated or analysed during this study are included in this published article.
